# Detection and Intervention of Clinically Masquerading Inferior Mesenteric Artery AVMs

**DOI:** 10.1155/2021/8854806

**Published:** 2021-05-28

**Authors:** Amir Pakray, Nolan Hayden, Farnoosh Sokhandon, Johnathan Olsen

**Affiliations:** ^1^Department of Diagnostic Radiology, Beaumont Hospital, Royal Oak, MI, USA; ^2^Central Michigan University College of Medicine, Mount Pleasant, MI, USA; ^3^Department of Diagnostic Radiology and Interventional Radiology, Beaumont Hospital, Royal Oak, MI, USA

## Abstract

We demonstrate a rare case of inferior mesenteric artery arteriovenous malformations leading to ischemic colitis in a 76-year-old female. Our patient presented with three months of nausea, vomiting, and diarrhea. Colonoscopy displayed diffuse mucosal vascular congestion while CTA and MRA displayed AVMs in the region of the IMA; however, cohesive clinical agreement on AVM from multiple specialties was difficult given its rare occurrence and nonspecific clinical, histopathologic, and directly visualized findings. The three noted dominant AVMs were eventually selected with coil and liquid embolization with successful cessation of symptoms and no major complications. Our discussion focuses on intervention and stressing the importance of radiologic findings, as IMA AVMs, rarely present as ischemic colitis and therefore can clinically masquerade as other etiologies.

## 1. Introduction

Ischemic colitis comprises over half of gastrointestinal ischemic insults and is most commonly noted in females, particularly over the age of 60 and with multiple comorbidities [1]. The most common and classic presentation of ischemic colitis is an acute onset abdominal pain, bloody stools, and leukocytosis. The patient in our case displayed the abovementioned demographics and some of the symptoms. Ischemic colitis can be classified into two categories of occlusive and nonocclusive etiologies. AVMs and AVFs fall underneath the umbrella of nonocclusive ischemic colitis; however, these are distinct entities with delineating factors, namely, that AVMs are typically congenital and AVFs are more often acquired [1]. Overall splanchnic vascular anomalies are rather rare with approximately 200 reported cases, and most notably the least common reported occurrence is in the IMA distribution, with hepatic and splenic territories more often encountered [1]. Therefore, the case we discuss highlights the rarest distribution of ischemic colitis from a vascular anomaly. Failure of undifferentiated embryonic vasculature regression is what leads to the typical imaging features of a dilated feeding artery, a nidus of tangled vessels and early draining veins [1]. Inferior mesenteric AVMs are typically accompanied by symptoms affiliated with portal hypertension such as ascites or variceal bleeding [2–4]. A smaller subset of patients display congestive ischemic colitis symptoms. Patients with congestive ischemic colitis due to inferior mesenteric AVMs present with abdominal pain, hematochezia, and diarrhea. Our case demonstrated this less common symptomatic array and presentation of AVM-induced ischemic colitis. As the clinical presentation of IMA AVMs can vary greatly, multiphase cross-sectional imaging plays a critical role in arriving at the correct diagnosis.

Current literature supports the use of MDCT angiography for initial evaluation of inferior mesenteric AVMs with subsequent colonoscopy in order to histopathologically exclude any masquerading disease [5]. Distribution and extent of disease is best assessed by utilizing MDCT angiography [1]. Although nonspecific, wall thickening or mural edema exhibited as a thickened hypodense appearance of the bowel wall is most commonly seen when ischemic colitis is encountered. Additional nonspecific findings include ascites and infiltration of the pericolonic mesenteric fat. All three of these imaging findings (bowel wall thickening, pericolonic mesenteric fat infiltration, and ascites) were noted on our case. Mucosal hypoperfusion and submucosal edema from arterial steal phenomenon and venous hypertension, respectively, explain the vascular mechanism behind congestive ischemic colitis [2, 6, 7].

Treatment is typically case-specific necessitating diagnostic consensus and clinical cohesion amongst various specialties including gastroenterology, vascular surgery, interventional radiology, and diagnostic radiology. Literature supports arterial embolization at the arteriovenous interface given its efficacy, noninvasive approach, and low complication rates [3, 8]. Athanasiou et al. suggest satisfying three criteria to prevent misdiagnosis and suboptimal intervention in cases suspected of ischemic colitis: first is identifying clinical signs of colonic ischemia, second is diagnostic imaging evaluation and confirmation of the etiology, in our case vascular malformation (ideally an angiogram), and third is direct visualization of a congested colonic mucosa via colonoscopy [9]. Larger and/or higher flow AVMs are directly associated with higher embolization complication rates [3, 8]. Embolization, although less invasive than surgical intervention, is not without risk. Embolization can cause organ ischemia and/or recurrence especially when more than one feeding vessel is identified [9]. The risk of nontarget embolization from particle migration has been noted to increase when a high-flow AVM displays a diameter larger than 8 mm [9]. Surgical intervention can potentially be curative; however, it is known to have higher risk of hemorrhage [9].

## 2. Case Presentation

**Figure 1:**
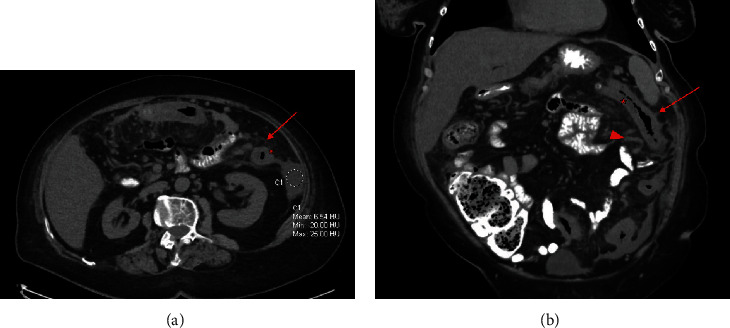
Initial presentation to the emergency department with diffuse abdominal pain and diarrhea. CT abdomen and pelvis with oral contrast only. Axial image (a) displays colonic mural thickening (star) and adjacent pericolonic fat stranding (arrow) most pronounced in the descending colon with ascites in the left paracolic gutter (circled). Coronal image (b) displays left hemicolon mural thickening (star) and adjacent pericolonic fat stranding (arrow) in the region of the IMA distribution. Suggestion of a prominent vascular structure is noted medial to the descending colon (arrowhead).

**Figure 2:**
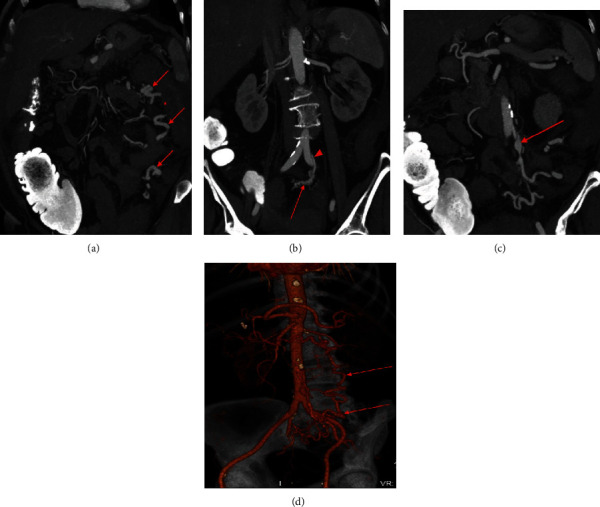
CTA of the abdomen and pelvis (early arterial phase images) 4 days after initial presentation. Maximum intensity projection (MIP) coronal image (a) displays prominent tortuous third-order branches of the IMA within the left abdomen adjacent to the descending colon (arrows). The descending colon appears thickened (star) with persistent pericolonic fat stranding. MIP coronal image (b) displays the superior rectal artery (arrowhead) leading to a tangle of vessels, the presumed nidus (arrow). MIP coronal image (c) exhibits aneurysmal dilatation of a venous outflow tract contiguous with outflow from the sigmoid territory (arrow). 3D volume rendered coronal oblique image (d) displays the prominent tortuous vascular structure in the inferior mesenteric vascular territory (arrow).

Our patient is a 76-year-old female with a past medical history of hypertension, type 2 diabetes mellitus, hypothyroidism, and gastroesophageal reflux disease, initially presenting to the emergency department (ED) with a three-month history of nausea, vomiting, and occasional diarrhea. She was evaluated by gastroenterology, and an outpatient EGD was performed showing a small hiatal hernia and atrophic gastritis. Within the next few weeks, she presented to ED with acute onset abdominal pain and worsening diarrhea which now appeared to be bloody. A CT scan of abdomen and pelvis was requested for the clinical diagnosis of ischemic colitis. Significant colonic wall thickening and pericolonic inflammatory stranding of the entire colon with sparing of the cecum, concerning for pancolitis with mild ascites, were noted on the initial CT scan (Figures 1(a) and 1(b)). The gastroenterology team was mostly concerned for noninfectious colitis, namely, ulcerative colitis or ischemic colitis, and boarded her for colonoscopy. Due to the patient's overall condition, comorbidities, and improvement of acute symptoms with supportive care, a colonoscopy was performed three days after admission when she was deemed stable. The results revealed pancolonic diffuse mucosal vascular congestion, pronounced in the rectosigmoid region, with a decreased mucosal vascular pattern. Random biopsies were performed which histopathologically revealed architectural distortion and paneth cell metaplasia, as seen in chronic colitis, without evidence of active colitis, granulomas, crypt abscesses, or dysplasia. The following day, a CTA of the abdomen and pelvis was obtained to evaluate for the possibility of an underlying vascular etiology. CTA showed that diffuse pancolitis slightly improved compared to the prior CT exam four days prior with a normal caliber patent abdominal aorta and proximal mesenteric vasculature. The CTA also displayed tortuous vascular lesions in the IMA territory with early filling veins concerning for AVMs (Figures 2(a)–2(d)). Given the rarity of AVM-induced ischemic colitis, patency of proximal mesenteric vessels, biopsy results suggesting chronic colitis, and overall clinical improvement with supportive therapy and steroids, the clinical suspicion favored ulcerative colitis. Given this presumed diagnosis, steroid therapy was continued and the patient was discharged in stable condition with recommendation for a follow-up in two weeks. At the time of follow-up, the patient had no abdominal symptoms and reported complete improvement.

**Figure 3:**
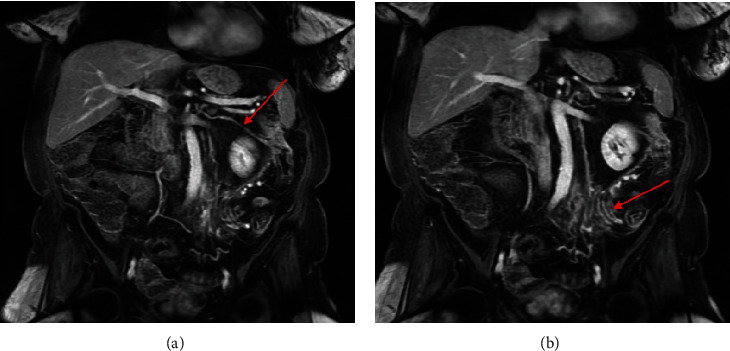
MRA of the abdomen with and without gadolinium 7 weeks after initial presentation. Coronal T1-weighted fat-suppressed VIBE late arterial phase postcontrast images display (a) a prominent marginal artery connecting the middle colic artery to the left colic artery (arrow) and also reveal (b) a cluster of early draining veins in the region of the sigmoid colon mesentery (arrow).

Approximately one month later, the patient presented to the emergency department with bilateral lower extremity edema and fatigue. Work-up for the edema revealed bilateral lower extremity DVTs, and the patient was placed on Lovenox. Shortly after, the patient developed several episodes of hematochezia and abdominal pain which prompted MRA of the abdomen, further strengthening the concern for AVMs. The MRA exhibited an inferior mesenteric artery distribution of multiple focal aneurysmal vessels in the left colonic mesentery (Figures 3(a) and 3(b)). Additionally, MRA displayed mild improvement in the patient's previously noted colitis.

**Figure 4:**
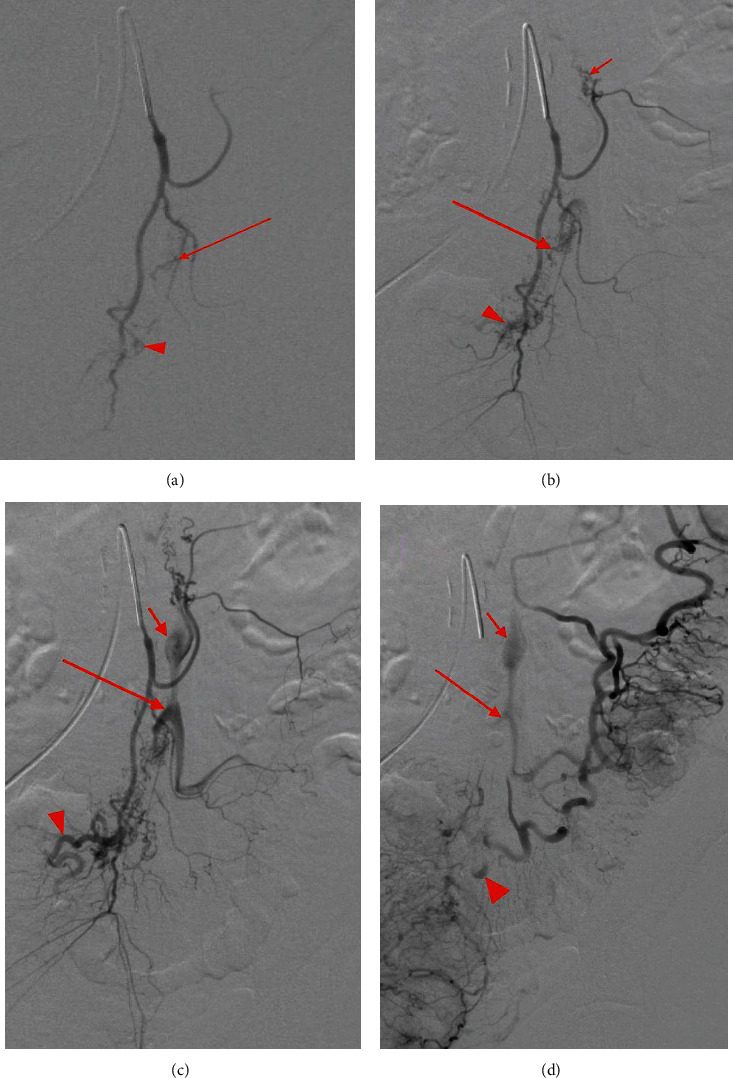
Mesenteric angiogram demonstrates subselection of the IMA in order of progressive filling which eventually displays three AVM niduses arising from the left colic artery, sigmoid artery, and superior rectal artery with respective dilated venous outflow tracts. Minimal opacification is seen in the presumed venous outflow tracts (a) of the AVM niduses at the sigmoid (red arrow) and superior rectal artery (arrowhead). Progressive opacification (b) is noted in the sigmoid (long arrow) and superior rectal niduses (arrowhead); opacification of the left colic nidus is demonstrated (short arrow). Ectatic venous outflow tracts of the left colic (c) (short arrow), sigmoid (long arrow), and superior rectal (arrowhead) AVM niduses are seen. Washout of the venous outflow tracts of the three AVM niduses (d) is demonstrated with similar labeling as (b).

**Figure 5:**
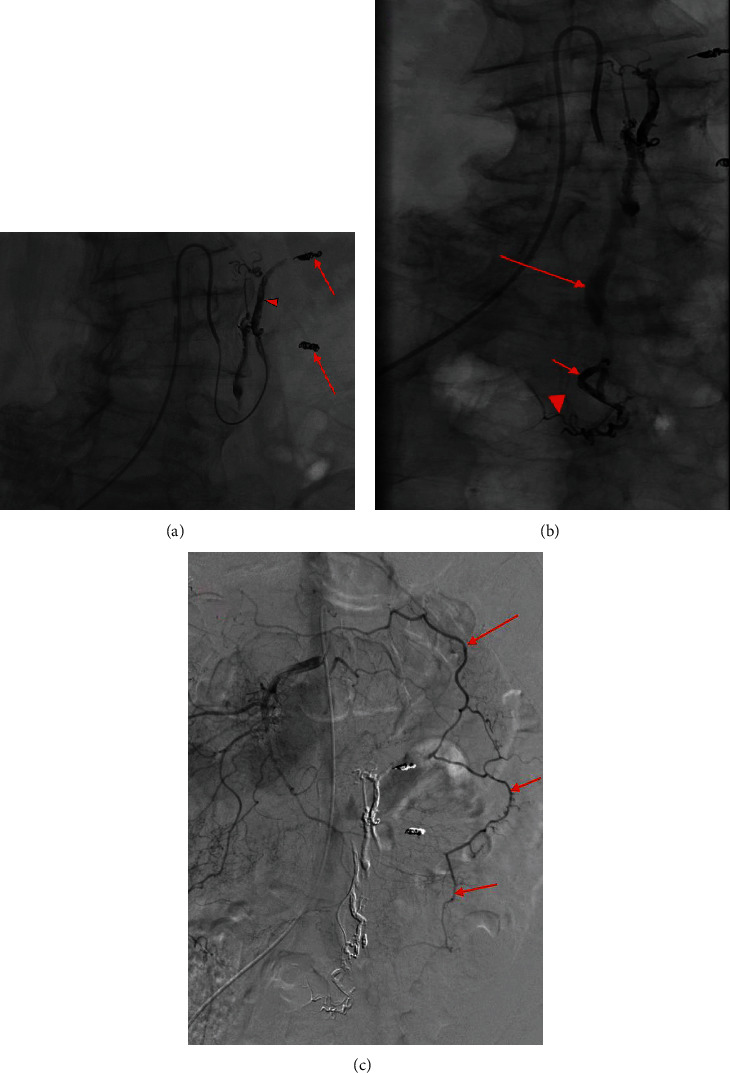
Intervention. (a) Coiling of left colic artery branches (arrows) prior to Onyx injection into the left colic artery (arrowhead) in order to prevent Onyx progression into the marginal artery. (b) Onyx embolization of the superior rectal artery (short arrow). Minimal filling of nontarget branches is also displayed (arrowhead). Onyx glue is seen through the AVM in the outflow vein of the left colic and superior rectal niduses. Inadvertent dissection of the IMA is demonstrated with stagnant contrast present in the IMA (long arrow). (c) Coils were deployed in two branches of the sigmoid artery to prevent Onyx filling of the marginal artery (arrows).

At this point, interventional radiology was consulted for a mesenteric angiogram. Arteriogram demonstrated a total of 3 AVMs, each individually supplied by branches of the IMA (Figures 4(a)–4(d)). The left colic artery was divided into two distal omental branches, which were coil embolized prior to Onyx liquid embolic instillation of the left colic artery (Figure 5(a)). The superior rectal artery was divided into two distal branches; both of which supplied the largest of the three AVMs, which was embolized with Onyx injection into the superior rectal artery proximal to both of the aforementioned branches (Figure 5(b)). The sigmoid artery, similar to the left colic, had two distal omental branches that were coil embolized before Onyx liquid embolic instillation (Figure 5(c)). There was an enlarged marginal vein draining into the middle colic vein with increased flow due to the AVMs. Subsequent SMA arteriogram displayed adequate flow to the left colon, sigmoid colon, and superior rectum from collaterals supplied by the marginal artery.

During postprocedural follow-up, the patient denied any abdominal pain, melena, or hematochezia. The patient demonstrated stability for over 1 year and was without any recurrence of abdominal symptoms.

## 3. Discussion

This is a novel case for several reasons and elucidates the importance of utilizing multiphasic cross-sectional imaging for an accurate diagnosis given the rare presentation. As previously noted, IMA distribution of AVMs is the least common distribution of splanchnic vascular anomalies. Furthermore, instead of presenting with the more common portal hypertension-associated symptoms, our patient presented with the less common presentation of ischemic colitis with hematochezia and diarrhea.

Initial confusion in diagnosis occurred for several reasons in our case. Retrospective evaluation of the initial ED non-IV contrast CT abdomen and pelvis did display tortuous vasculature in the IMA distribution; however, given that no IV contrast was given, this finding can easily be overlooked. Colonoscopy showed mucosal vascular congestion, and histopathology revealed architectural distortion seen in chronic colitis; thus, an acute ischemic colitis was considered less likely. The subsequent CTA of the abdomen and pelvis did however delineate the vascular malformations very well. However, given the rarity of AVM-induced ischemic colitis and pathology findings, clinical management of chronic colitis was continued. This highlights the importance of scrutinizing the associated intestinal vasculature when bowel pathology is demonstrated. Multimodality multiphasic cross-sectional imaging (CTA and MRA) built a very strong diagnosis of IMA distribution AVMs, allowing visualization of the niduses in the arterial and venous phase along with any underlying vascular pathology that could preclude intervention. Mesenteric angiogram further confirmed the suspected three niduses in the IMA distribution. Additionally, the angiogram allowed the interventional team to deem the AVMs amenable to liquid and coil embolization as they did not appear too high flow or large for intervention.

This case report is limited due to the lack of postprocedural imaging which would allow for thorough evaluation of the vascular anatomy and collateralization, serving as a beneficial baseline if future complications were to arise. However, in our case, the patient had no complications in the postprocedural course, and therefore, imaging was not clinically indicated. The multimodality imaging of this case report (CT, CTA, MRA, and angiogram) allows for wholesome diagnostic and interventional evaluation and augments our understanding of a rare vascular anomaly.

Ischemic colitis secondary to AVMs can be strongly suggested and diagnosed on imaging, and with multispecialty diagnostic consensus can lead to early treatment and fewer complications. This elucidates that the imperative role of radiology can play in suggesting a firm diagnosis and providing appropriate intervention.

## Data Availability

Full anonymized datasets (DICOM images, aside from the provided images in the case report) used to support the findings of this study are available from the corresponding author upon request.
